# Genetic Structure of Wild Bonobo Populations: Diversity of Mitochondrial DNA and Geographical Distribution

**DOI:** 10.1371/journal.pone.0059660

**Published:** 2013-03-27

**Authors:** Yoshi Kawamoto, Hiroyuki Takemoto, Shoko Higuchi, Tetsuya Sakamaki, John A. Hart, Terese B. Hart, Nahoko Tokuyama, Gay E. Reinartz, Patrick Guislain, Jef Dupain, Amy K. Cobden, Mbangi N. Mulavwa, Kumugo Yangozene, Serge Darroze, Céline Devos, Takeshi Furuichi

**Affiliations:** 1 Primate Research Institute, Kyoto University, Inuyama, Japan; 2 Lukuru Wildlife Research Foundation, Kinshasa, Democratic Republic of Congo; 3 Bonobo and Congo Biodiversity Initiative, Zoological Society of Milwaukee, Milwaukee, Wisconsin, United States of America; 4 African Wildlife Foundation, Kinshasa, Democratic Republic of Congo; 5 Research Center for Ecology and Forestry, Ministry of High Education and Scientific Research, Mabali, Democratic Republic of Congo; 6 World Wide Fund for Nature, Kinshasa, Democratic Republic of Congo; 7 Department of Anthropology, Emory University, Atlanta, Georgia, United States of America; University of Florence, Italy

## Abstract

Bonobos (*Pan paniscus*) inhabit regions south of the Congo River including all areas between its southerly tributaries. To investigate the genetic diversity and evolutionary relationship among bonobo populations, we sequenced mitochondrial DNA from 376 fecal samples collected in seven study populations located within the eastern and western limits of the species’ range. In 136 effective samples from different individuals (range: 7–37 per population), we distinguished 54 haplotypes in six clades (A1, A2, B1, B2, C, D), which included a newly identified clade (D). MtDNA haplotypes were regionally clustered; 83 percent of haplotypes were locality-specific. The distribution of haplotypes across populations and the genetic diversity within populations thus showed highly geographical patterns. Using population distance measures, seven populations were categorized in three clusters: the east, central, and west cohorts. Although further elucidation of historical changes in the geological setting is required, the geographical patterns of genetic diversity seem to be shaped by paleoenvironmental changes during the Pleistocene. The present day riverine barriers appeared to have a weak effect on gene flow among populations, except for the Lomami River, which separates the TL2 population from the others. The central cohort preserves a high genetic diversity, and two unique clades of haplotypes were found in the Wamba/Iyondji populations in the central cohort and in the TL2 population in the eastern cohort respectively. This knowledge may contribute to the planning of bonobo conservation.

## Introduction

Bonobos (*Pan paniscus*) live on the left bank of the Congo Basin and are separated from other *Pan* populations by the Congo River. The monophyletic origin of bonobos in great apes is supported by recent molecular phylogenetic studies [1], [2]. The divergence time of the bonobo from the chimpanzee (*Pan troglodytes*) has been estimated to be about 1 million years ago (Ma) [3]–[5].

**Figure 1:**
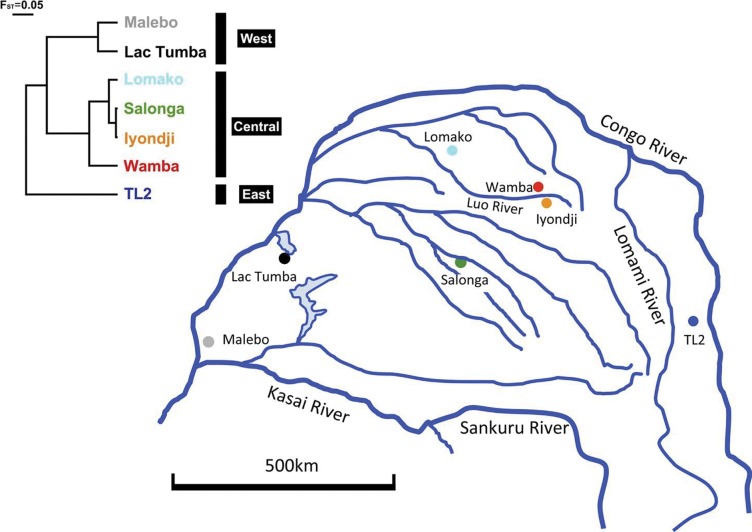
Study area and a population tree. Right map shows geographical location of study populations in DRC. Rivers indicated here are based on limnological study [42]. Left is a population tree constructed by UPGMA method with net population distances estimated from calculation of F_ST_ distances.

Concerns have been expressed that increased logging roads and deforestation will progressively lead to fragmentation of bonobo habitat [6]. Under such circumstances, understanding the genetic structure and gene flow among bonobo populations is of utmost importance for planning adequate conservation programs that preserve genetic diversity for the future. A previous study identified the Lomami River, a large tributary of the Congo River, as a barrier to gene flow among populations [7]. Two mitochondrial DNA (mtDNA) clades have been found in five wild bonobo populations [7], and a third clade of undefined wild origin has been reported in captive bonobos [1]. However, our knowledge about the genetic structure in the entire bonobo habitat range is limited. In order to define the geographical distribution of haplotypes, we collected samples at seven sites that covered a broader range than was the case in previous studies of bonobos (Figure 1), and performed genetic assessments to characterize the molecular phylogenetic features among mtDNA haplotypes and genetic differentiation within and among study populations.

To examine the intraspecific genealogy in a phylogeographic framework, we collected a total of 376 fecal samples from seven populations (Fig. 1), and for 136 effective samples, we compared complete sequences of noncoding regions in the mtDNA. In Africa, two evolutionary effects for diversification within a species have been reported in primates: riverine barriers [7] and Pleistocene refugia [8]. Additionally, a combined effect has been reported [9]. We investigated the evolutionary history of the genetic structure of bonobo populations by examining genetic differentiation by distance and rivers as a barrier to gene flow.

## Results and Discussion

### MtDNA Haplotypes

**Figure 2:**
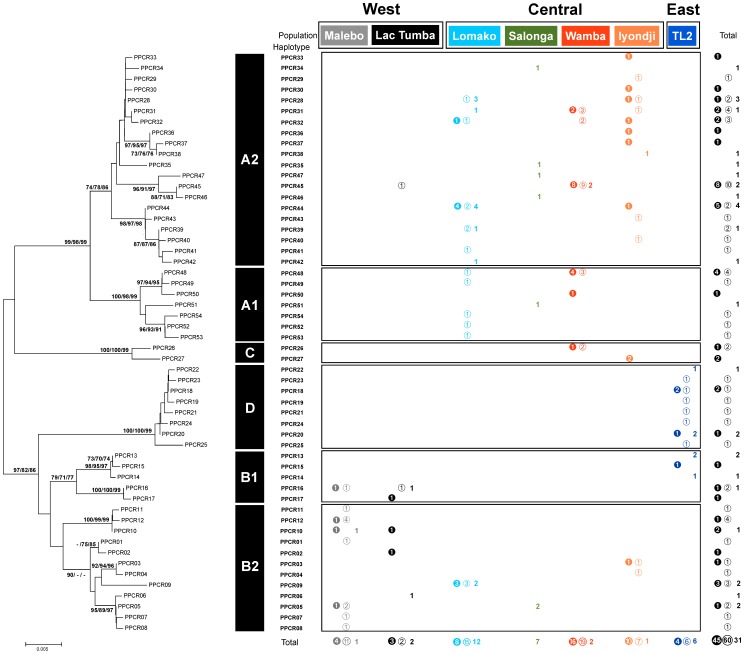
Molecular phylogeny of haplotypes and their distribution in study populations. Left is a tree constructed by neighbor-joining (NJ) method. Three numbers on a tree path indicate percent bootstrap values (1,000 replications) obtained from statistical assessments by neighbor-joining (NJ), maximum likelihood (ML) and maximum parsimony (MP) algorithms in order. Branches corresponding to partitions reproduced in less than 70% bootstrap replicates were collapsed. Each of black bars aside the tree shows a mtDNA clade inferred from the cluster analyses. Right score illustration summarized distribution of mtDNA haplotypes in study populations. Numbers with closed, open circles and without circle mean the observed number of male, female and sex-unknown samples, respectively, for each study population with different color.

Gblock sorting of 1128 nucleotide sites in the initial alignment extracted 1121 sites (99%) consisting of three selected blocks of flanking positions. Consequently, we distinguished 54 mtDNA haplotypes in all the samples. MtDNA haplotypes were locally clustered in the bonobo samples from the Democratic Republic of the Congo (DRC), in which 45 haplotypes (83%) were locality-specific (autoapomorphic) and only 9 (17%) were shared (synapomorphic) by two or three populations (Figure 2). The proportion of haplotypes shared with other populations was high in the Wamba (4/6; 67%) and Lac Tumba populations (3/6; 50%), intermediate in the Malebo (3/8; 38%), Lomako (5/13; 38%), Iyondji (4/15; 27%), and Salonga populations (1/6; 17%), and low in the TL2 population (0/11; 0%), suggesting temporal isolation of the TL2 population in the eastern periphery.

Clustering analyses revealed six groups of haplotypes (haplogroups) in this study. Three of these groups were named A, B, and C clades in previous studies [1], [7] and we newly identified D clade in this study. Since we detected two new subgroups in both the A and B clades, we renamed the new clades as A1, A2, B1, and B2, in addition to clades C and D (Figure 2). Component haplotypes of the A1, A2, B1, and B2 clades were shared by more than three study populations but those of C and D were found only in the Wamba/Iyondji and TL2 populations, respectively. Bonobo females transfer among groups whereas males stay in their natal group for life [10], [11]. The existence of certain haplotypes in male samples suggested that those haplotypes had been maintained over generations rather than representing occasional transfer of females, because a haplotype is found in male samples only when females who brought the haplotype produced male offspring.

The results of clustering suggested that the observed clades were evolutionarily related to each other, with a substantial number of nucleotide substitutions, in which the mean number of pairwise haplotype differences between the clades (36.03±7.69) was 4.7 times larger than that within each clade (7.74±2.61) (Table S2).

The unique haplotypes of the C clade have been reported previously, but their geographical distribution in the wild was not identified [1]. This study confirmed distribution of haplotypes in the C clade in the Wamba and Iyondji populations. One of the haplotypes of the C clade that was previously found in an exported bonobo (Accession Number AF176762 [12]) was found in the Wamba population (PPCR26 type), but several other haplotypes in the same clade that were reported in individuals in captivity (AF137491 [1], GU189665 as “PP56” and GU189670 as “PP69” [1]) were absent in either the Wamba or Iyondji populations. This suggests that the distribution of bonobos having the C clade haplotypes may have a broader range than that confirmed in this study.

We named the sole clade consisting of autoapomorphs in TL2 as D clade. Their related sequences have previously been reported (AJ829464–AJ829466 as “E_3_ to E_5_” [7]), and there was one case of type matching between a sample from this study and one from the same report (PPCR24 and AJ8294564 as “E3” [7]). However, this isolated D clade was not found in previous studies with full confidence probably due to comparison based on the short sequence lengths. Thus the component haplotypes of D clade have already been reported and we confirmed that these haplotypes comprise an independent clade.

Application of long sequence reading revealed a finer image of mtDNA phylogeny in bonobos than that in a previous study [7]. However, geographical distribution of mtDNA haplotypes needs future assessment because numbers of available samples were particularly small (n = 7) for Salonga and Lac Tumba in this study.

### Genetic Diversity within Populations

The diversity of mtDNA in terms of haplotype diversity, mean number of pairwise differences, and nucleotide diversity varied among populations (Table 1). Although the sample size differed among populations, mtDNA diversity showed no significant correlation with sample size (r = –0.359; 0.4<*p*<0.5 for the mean number of pairwise differences, r = –0.360; 0.4<*p*<0.5 for nucleotide diversity). The Malebo, TL2, and Wamba populations had relatively lower diversity. The Wamba population revealed the smallest estimate of haplotype diversity (0.694), suggesting a decline in the variety of haplotypes in this population. The Malebo and TL2 populations, inhabiting the western and eastern periphery of the species’ distribution, respectively, also showed lower estimates of the mean number of pairwise differences and nucleotide diversity than other study populations. In contrast, populations in the central region (except for the Wamba population) were characterized by abundant and diverse mtDNA variations.

**Table 1:** Genetic diversity of mtDNA haplotypes within seven populations of bonobos in DRC.

Population	Malebo	LacTumba	Lomako	Salonga	Wamba	Iyondji	TL2
No. of samples	16	7	35	7	37	18	16
No. of haplotypes	8	6	13	6	6	15	11
Polymorphic sites	34	59	60	60	56	65	36
Haplotype diversity (mean±sd)	0.875±0.059	0.952±0.096	0.861±0.038	0.952±0.096	0.694±0.067	0.980±0.024	0.942±0.041
Mean no. of pairwise difference (mean±sd)	12.954±6.161	24.990±12.531	22.084±9.969	28.704±14.344	16.837±7.665	22.167±10.249	14.169±6.709
Nucleotide diversity (mean±sd)	0.0116±0.0062	0.0223±0.0128	0.0197±0.0099	0.0256±0.0147	0.0150±0.0076	0.0198±0.0102	0.0126±0.0067

The genetic diversity of populations usually declines from the center of a geographical range to the periphery [13]. The mechanism that generates this pattern is not clear, but it could be caused by peripheral populations being small and isolated. The differences in the genetic diversity among the study populations matched this pattern, except for the low diversity in the Wamba population in the central region. The Wamba population has long been isolated from other populations by roads and human habitation [14]. Furthermore, during times of political instability and two civil wars (1991–2002) in the DRC (formerly known as Zaïre), the Wamba population decreased in size and some groups were missed [14], [15]. The lower genetic diversity in the Wamba population may have been caused by these factors. In contrast to the Malebo and TL2 populations, which showed similarly low diversity, the Wamba population showed an imbalance among diversity indexes, in which the number of haplotypes (haplotype diversity) was relatively small compared to the mean number of pairwise differences and nucleotide diversity (Table 1). This imbalance likely reflects an unbalanced decline between the number of genes and gene heterogeneity typically known as “heterozygosity excess” (in diploid systems) in the initial phase of a population bottleneck [16].

### Genetic Differentiation among Populations

The population distances measured by pairwise (F_ST_) genetic distances and the statistical test results are presented in Table S3. Many of the distance estimates were significantly large, but not for the differences in populations such as Malebo vs. Lac Tumba (F_ST_ distance = 0.111, *p* = 0.09), Salonga vs. Lac Tumba, Lomako, Wamba, Iyondji (F_ST_ distances = 0.004–0.191, *p* = 0.05–0.33), and Lomako vs. Iyondji (F_ST_ distance = 0.046, *p* = 0.09). We constructed a population tree using the unweighted pair group method with arithmetic mean (UPGMA) using net pairwise F_ST_ distances to find the proximity of mtDNA variations among study populations. The results revealed three clusters of bonobos in the DRC, corresponding to the Malebo and Lac Tumba cohort in the west region; the Lomako, Wamba, Iyondji, and Salonga cohort in the central region; and TL2 as the sole cohort in the east region (Figure 1).

Genetic assessment of the population structure using analysis of molecular variance (AMOVA) revealed a slight difference in the total variation partition between the two population categories: we found 40.8% of the mtDNA variation among the seven study populations and 48.0% variation among the three cohorts (Table 2). Therefore, the partition of genetic variation among the seven populations is well represented by the three cohorts. In a pairwise F_ST_ population comparison, the genetic distance between central and east (0.5334) was larger than those observed between west and central (0.4281) and between west and east (0.4731). Based on the genetic distances, the east region could be regarded as being more distantly related to the central than the west. This conspicuous feature of the genetic relationships among the three population cohorts gives serious cause for reconsidering the hypothesis that the riverine barrier to individual migration was a significant factor in the evolution of this species. [7].

**Table 2:** Comparison of geographical structure of populations by assessments with AMOVA.

Comparison		df	SSD	Variation %
Seven study populations	Among populations	6	717.13	40.82
	Within populations	129	1129.17	59.18
Three population cohorts (west, central, east)	Among cohorts	2	545.09	47.95
	Within cohorts	133	1244.77	52.05

### The Effect of a Riverine Barrier on Genetic Variation among Populations

**Figure 3:**
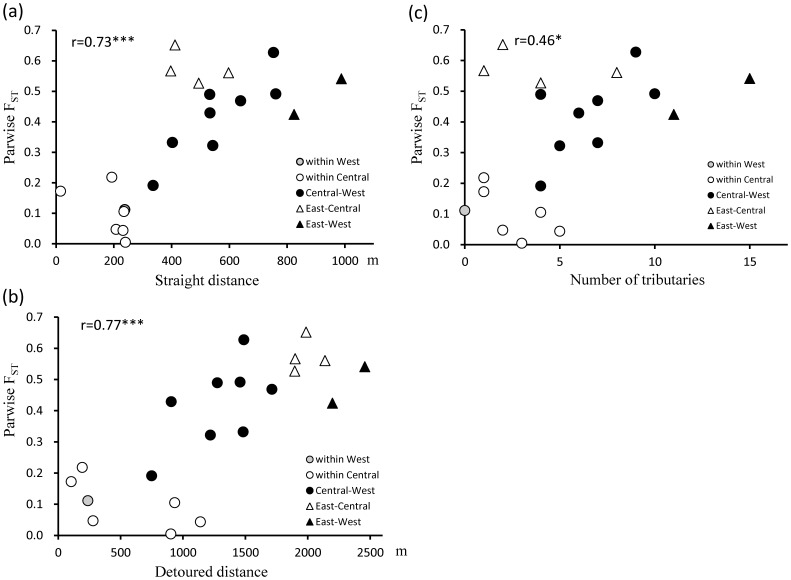
Relation between genetic distance (F_ST_) and geographical indices. Each pair of seven populations, in all 21 pairs, is dotted as a different symbol according to combination of cohorts. (a) Geographical distance between two populations was measured as a straight line. (b) Geographical distance was measured by detouring headwater of big tributaries or lakes. (c) Number of tributaries on the straight-line between two populations. *p<0.05, ***p<0.001.

The differentiation process of mtDNA among the study populations was examined by comparing three geographical factors: straight distance, detoured distance, and number of tributaries (see Materials and Methods). The genetic distance (F_ST_) among the populations showed significant coefficients of correlation with all the geographical indices (Figure 3). The number of tributaries showed the lowest coefficient of correlation with the genetic distance. In contrast, the detoured distance showed a higher coefficient of correlation than the straight distance, although the difference in the coefficients between these two factors was negligible. Therefore the riverine effect was not concluded by this analysis.

Table 3 compares correlations between F_ST_ and the three types of geographical factor from each study site to other sites. Straight distance showed significant positive correlations with F_ST_ in four of the seven populations. In contrast, detoured distance showed significant correlations in only two populations (Wamba and Iyondji) and the number of tributaries showed a significant correlation with one population (Malebo). Although not statistically significant, the TL2 population consistently showed negative coefficients in the comparison, which suggests that the factors influencing the genetic distance between TL2 and other populations differed from those influencing the other six populations. When TL2 was excluded from the calculations, statistically significant correlation coefficients were noted in five populations for straight distance, none for detoured distance, and only one population (Malebo) for the number of tributaries (Table 3).

**Table 3:** Correlation between genetic distance (F_ST_) and geographical distance from a specific area to other areas.

Area	To other six areas (n = 6)						To other five areas (TL2 was removed from calculations) (n = 5)					
	r (with straight distance)		r (with detoured distance )		r (with number of tributaries)		r (with straight distance)		r (with detoured distance )		r (with number of tributaries)	
TL2	−0.58	ns	−0.32	ns	−0.55	ns						
Iyondji	0.68	ns	**0.81**	*	0.37	ns	0.82	ns	0.68	ns	0.85	ns
Wamba	**0.82**	*	**0.84**	*	0.49	ns	**0.93**	*	0.76	ns	0.78	ns
Salonga	**0.96**	**	0.60	ns	0.41	ns	**0.97**	**	−0.35	ns	0.76	ns
Lomako	**0.92**	*	0.79	ns	0.73	ns	**0.89**	*	0.65	ns	0.61	ns
Lac Tumba	0.78	ns	0.81	ns	0.64	ns	**0.88**	*	0.84	ns	0.59	ns
Malebo	**0.87**	*	0.79	ns	**0.83**	*	**0.95**	*	0.88	ns	**0.94**	*

* p<0.05, **p<0.01.

To compare single-factor and multifactor models, we calculated Akaike’s information criterion (AIC) based on 21 pairs (Figure 3) for single-factor models involving each of the geographical factors (Table 4) and a two-factor model involving the straight distance and number of tributaries (Table 5). Detoured distance had the smallest AIC among the three single factors, similar to the result observed with the correlation analysis (Figure 3). However, the calculations based on 15 pairs (when TL2 was excluded) indicated that straight distance showed the least AIC (Table 4). By two-factor analysis, the straight distance had a greater effect on genetic distance than did the number of tributaries (Table 5). The AIC of this model was not smaller than the single-factor model involving straight distance in the analysis of the 15 pairs (Table 4). Thus, the riverine barrier appeared to have a weak effect on gene flow among populations, except for the Lomami River, which separates TL2 from other populations.

**Table 4:** Calculations of AIC using GLM for single factor models.

Factor	All areas (n = 21)			When TL2 was removed (n = 15)		
	t	p	AIC	t	p	AIC
Straight distance	**4.7 (+)**	**0.000175**	−16.74	**6.6 (+)**	**0.0000169**	−23.42
Detoured distance	**5.2 (+)**	**0.0000473**	−19.51	**3.1 (+)**	**0.00905**	−9.49
Number of tributaries	**2.3 (+)**	**0.03571**	−5.78	**3.8 (+)**	**0.00215**	−12.6

F_ST_ was used as a response variable and Gaussian (identity) was used as a family (link function). Signs in parenthesis mean direction to increase F_ST_.

**Table 5:** Calculations of AIC using GLM for two-factor models.

Factors	All areas (n = 21)		When TL2 was removed (n = 15)	
	(AIC = −23.21)		(AIC = −21.68)	
	t	p	t	p
Straight distance	**5.2 (+)**	**0.000056**	**3.6 (+)**	**0.0035**
Number of tributaries	−**3.0 (**−**)**	**0.00784**	−0.5 (−)	0.6547

F_ST_ was used as a response variable and Gaussian (identity) was used as a family (link function). Signs in parenthesis mean direction to increase F_ST._

The riverine barrier effect was highlighted for gene flow among bonobo populations in a previous study, and the Lomami River, separating the TL2 population from others, was considered the sole barrier to migration [7]. According to the riverine barrier hypothesis, however, it is difficult to explain why the east cohort showed closer proximity to the west cohort than to the central cohort. In addition, the lower genetic distances among the Iyondji, Salonga, and Lomako populations (central cohort) than between the Lac Tumba and Malebo populations (west cohort) suggest that the riverine barrier had only a weak effect on genetic distance in the central region. Although all of the large tributaries can be regarded as barriers at present, they might not have functioned as effective isolation barriers in geological time.

An alternative explanation for the observed differentiation in bonobo populations is that the genetic differentiation and historical fragmentation may have been caused by the locations of refugia during the Pleistocene. Several studies have suggested that the location of forest refugia in the Congo Basin at the Last Glacial Maximum (LGM; circa 18,000 years ago) was in the central parts of the southern Congo Basin [17], [18], whereas other studies have described riparian refugia along the Congo River and its main tributaries [19]–[21]. Wherever the refugia locations, the present data suggest that the bonobo population dispersed from a limited area along with the expansion of the forest. The barrier effect of rivers during dry periods was probably reduced by their decreased width [22]. Therefore, the evolutionary history of populations during the Pleistocene suggests that present-day tributary systems have had only a small effect on the genetic structure of current bonobo populations.

Regarding the Lomami River, neither the detoured distance nor the number of tributaries showed a positive correlation coefficient with genetic distance from the TL2 population (Table 3). This observation might indicate the isolation of the TL2 population from other populations for a certain geological time, rather than low occurrence of gene flow between TL2 and other populations due to detouring caused by the Lomami River. TL2 shared no haplotypes with other populations and showed quite different coefficients in the correlation analysis (Table 3). Furthermore, the haplotypes of the D clade were found only in this region (Figure 2). Nevertheless, it contained specific haplotypes of the B1 clade coupled with the west cohort. Future studies will be required to elucidate how the B1 haplotypes are shared between east and west regions (Figure 2). These results might be explained not only by prevention of individual migration by existing riverine networks but also by historical separation of habitats associated with paleoenvironmental changes. The TL2 population might have inhabited another refugium at the LGM between the Congo and Lomami rivers [17], [23].

Present-day rivers as barriers to gene flow could not fully explain the genetic structure of bonobo populations confirmed in this study. The geographical pattern of the bonobo genetic structure seems to have formed over hundreds of thousands of years. After bonobos and chimpanzees diverged about 1 Ma [3]–[5], the common ancestor of extant bonobos lived until as recently as 500,000 years ago [1], [24]. Even at 500,000 years ago, differentiation of some clades of bonobos occurred long before the LGM (Figure 2). This means that bonobos were affected not only by forest reduction in the LGM but also by climate changes during the Pleistocene, such as the glacial–interglacial pattern. More information on paleoenvironmental changes in the Congo Basin during the Pleistocene is required to elucidate the genetic structure of bonobo populations.

### Conservation of Bonobos

In this study, we classified the bonobo populations in the DRC into three cohorts in different localities (Figure 1). Strong segregation of the cohorts was supported by the observed mtDNA diversity, and they can be regarded as potential evolutionarily significant units in conservation applications [25]. In addition, the geographical distribution of the six clades might reflect differences in evolutionary backgrounds among study populations. To define the species-level diversity of bonobos further, future studies should include samples collected from more locations and investigate different genetic markers.

In the conservation of great apes, it is important to prioritize areas with high genetic diversity and to preserve unique haplotypes. Therefore, the current study showing the distribution of haplotypes in a broad bonobo habitat will hopefully contribute to the planning of bonobo conservation.

## Materials and Methods

### Sampling Populations and Methods

DNA sampling was performed at natural sites in bonobo habitats in the DRC from July 2010 to March 2012. We collected fecal samples of wild bonobos from seven distinct populations in the DRC (Figure 1). Basic information regarding each research site has been described for various bonobo populations, including the TL2 population in the south [26], Iyondji and Wamba [15], [27], Salonga [28], Lomako [29], and Lac Tumba and Malebo [30]. Most bonobo habitats are covered with thick tropical rainforest. The fecal samples collected in Malebo and areas south of the TL2 population, however, were found in savannah-forest mosaic vegetation. Few geographical barriers to migration were present among the study populations, except for some tributaries of the Congo River or deep swampy forest.

When we found feces under nests, we estimated the freshness of the nest (most were less than a day old), the number of nests, and latitude and longitude using a global positioning system (GPS). For well-habituated groups, main parties were followed from nest to nest and feces were collected when bonobos defecated during direct observation. The sampling never interacted or interfered with bonobos because samples were obtained non-invasively under the nested tree or directly from the nest after leaving of the host.

We also estimated the size (large, medium, small) and hardness (solid, intermediate, soft) of the feces to check health status and to estimate the recovery rate of the mtDNA. To estimate genetic diversity within a population, we collected fecal samples from two or more groups for each population. The sampling procedure was as follows. First, a dry cotton swab was rolled on the surface of the fecal sample as extensively as possible. Second, the end of the cotton swab was washed in lysis buffer to shake the feces off the cotton swab. This swabbing procedure was performed at least three times to collect cells. Third, each fecal sample was turned over and steps 1 and 2 of the collection process were repeated using the other side of the cotton swab. Fourth, the tube caps were fastened and the sample number was marked on each tube.

The Scientific Authority for CITES and National Scientific Committee for the Worldwide Heritage of UNESCO in DRC has confirmed that we can publish the results obtained from the fecal samples of bonobos carried out from DRC as far as we have research permission that includes the permission to use those samples. Research Permissions during this study were issued by following authorities: 024/ICCN/BP-MA/2010 (for TL2), 051/ICCN/DG/ADG/KV/2011 (for Lomako), 1577/ICCN/ADG/ANG/DG/2008 (for Salonga) were given by the Institut Congolais pour la Conservation de la Nature (ICCN). MIN.RS/SG/002/2010 (for Iyondji), MIN.RS/SG/003/2010 (for Wamba), 008//MINRS/CREF/MAB/DG/01MNIK/2011 (for Lac Tumba) were given by Ministère de la Recherche Scientifique (MIN). 001/CREF/2012 (for Malebo) was given by the Centre de Recherche en Ecologie et Foresterie (CREF).

### DNA Samples

A total of 376 fecal samples were collected for DNA study in the Democratic Republic of Congo between July 2010 and February 2012. DNA extraction was performed using a proprietary procedure combining a sampling lysis buffer [31], removal of the potential PCR inhibitors such as bile salts and bilirubin with starch [32], and a commercially available DNA cleanup system with a silica membrane (Wizard SV Gel and PCR Clean-Up System; Promega, Madison, WI, USA) (details are presented in Table S1). The SDS lysate of gut cells was preserved in a tube at ambient temperature until DNA extraction (maximum of 6 months). Qualification of purified DNA samples was made quantitatively [33] or qualitatively by an electrophoretic procedure (Table S1). Multiple sampling from the same individual was inspected as much as possible by genotyping with 10 microsatellite markers (data not shown), but this evaluation was incomplete for some samples due to difficulty with the genotyping. Among the collected samples, we failed to sequence the target mtDNA region for 114 samples due to low DNA quality or recovery and confirmed 126 cases of multiple sampling. Finally, we judged that 136 DNA samples were taken from different individuals and subjected them to comparative analysis in this study.

### DNA Sequencing

A complete sequence of mtDNA noncoding region was determined from each fecal sample. A DNA fragment spanning the target region was amplified and sequenced using the seven primers listed in Table S1. PCR amplification was performed with a high-success rate DNA polymerase KOD FX (Toyobo, Osaka, Japan) and sequences were read manually by direct sequencing. False readings caused by nuclear mitochondrial DNA (Numt) were verified by aligning the obtained sequence reads with published data. Obtained sequence data were deposited in DDBJ/EMBL/GenBank databases (Accession Numbers AB780372–AB780425). The data were subjected to size adjustments by sorting with Gblocks [34] under the default stringent condition for haplotyping and subsequent phylogenetic or population analyses.

### Molecular Data Analysis

Haplotypes were defined by multiple alignments with ClustalX ver. 2.1 [35] for the sorted sequences. The Tamura-Nei model [36] was assumed in the computation of evolutionary distance. Molecular phylogenetic relations were inferred by using neighbor-joining (NJ), maximum likelihood (ML), and maximum parsimony (MP) algorithms as implemented in MEGA version 5 [37]. Haplogroups were classified based on the clusters resolved in tree constructions with statistical verification of 1,000 replications of bootstrap.

### Population Data Analysis

The mtDNA diversity within populations was estimated in terms of haplotype (gene) diversity, mean number of pairwise difference, and nucleotide diversity [38] using the program Arlequin version 2.000 [39]. Genetic differentiation between populations was quantified from calculations of intra- and interpopulation distances with pairwise F_ST_ distance [40] and average pairwise difference [38].

### Estimation of the Riverine Effect on Genetic Distance among Populations

According to the riverine barrier hypothesis, the genetic similarity between populations separated by a river should be higher in the headwaters (where the river is narrower) than in its lower parts [41]. We compared three geographical indices against genetic distance. Straight distance indicated the length of the straight line linking two study sites. Detoured distance indicated the length of a bent line that linked two study sites. The bent line had not to cross any large tributary (Figure 1), and to detour until the headwater to reach the opposite bank [7]. The center of the location of each population was roughly estimated as the center of gravity for the sampling places in each study population. These two types of geographical distances were measured using QGIS (ver. 1.8.0). The number of tributaries indicated the number of times rivers crossed the straight line on the satellite map (Google Earth). A riverine was regarded as a tributary only when it was estimated to be at least as wide as the Luo River on the satellite map. The width of the Luo River between the Wamba and Iyondji populations currently prevents bonobos from moving to the opposite bank. The number of tributaries between two populations was greater than the number of tributaries expected from Figure 1 in most cases.

The analyses were performed with R and JMP (SAS Institute, Cary, NC, USA). For the dataset of genetic distance (pairwise F_ST_), a normal distribution was not rejected. (Kolmogorov-Smirnov test, D = 0.16, *p* = 0.57, n = 21). Correlations were tested using Pearson’s correlation test. A generalized linear model (GLM) was used for calculations of AIC to estimate whether tributaries of the Congo River influenced genetic distances.

## Supporting Information

Table S1 **Summary of DNA experiments.**
(TIF)

Table S2 **Comparison of six clades of haplotypes with** **mean number of pairwise haplotype differences.** Mean number of pairwise haplotype differences was compared within and between clades shown in Figure 2. Values of the diagonal indicate average number of pairwise differences within clades. Those above the diagonal are average number of pairwise differences between clades and below the diagonal are corrected average pairwise differences. Estimates were obtained assuming Tamura-Nei mutation model using the software Arlequin version 2.000 (Schneider et al. 2000). Numbers in parentheses give the number of haplotypes in each clade.
(TIF)

Table S3 **Comparison of population distances with results of test for their statistical significance.** Values below the diagonal indicate estimates of population pairwise F_ST_ calculated assuming Tamura-Nei mutation model. Values above the diagonal indicate P values of permutaion test (n = 1,023) for the null hypothesis of F_ST_ = 0 by the software Arlequin version 2.000 (Schneider et al. 2000).
(TIF)
